# Parent Joint AB Blood Group Is Associated With Clinical Outcomes of *in vitro* Fertilization and Intracytoplasmic Sperm Injection Treatment in Chinese Women

**DOI:** 10.3389/fmed.2022.813781

**Published:** 2022-05-04

**Authors:** Xiao Bao, Feifei Zhao, Hao Shi, Zhiqin Bu, Yuling Liang, Yingpu Sun

**Affiliations:** ^1^Center for Reproductive Medicine, The First Affiliated Hospital of Zhengzhou University, Zhengzhou, China; ^2^Henan Key Laboratory of Reproduction and Genetics, The First Affiliated Hospital of Zhengzhou University, Zhengzhou, China

**Keywords:** clinical outcomes, *in vitro* fertilization (IVF), intracytoplasmic sperm injection (ICSI), husband/wife joint ABO blood type, success rate

## Abstract

**Background:**

A number of publications have examined the relation between blood group and female infertility including ovarian reserve, recurrent miscarriage, and live birth. However, there is a lack of literature investigating joint mother/father ABO blood type in a large cohort. This study aimed to investigate the association between couple combinations for ABO blood groups and assisted reproductive technology (ART) outcomes in patients undergoing *in vitro* fertilization (IVF)/intracytoplasmic sperm injection (ICSI).

**Methods:**

This retrospective cohort study included 30,717 couples who underwent IVF cycles between 2010 and 2019. The clinical outcomes of IVF treatment were the primary outcome. History of spontaneous miscarriage, embryo quality, and birth sex, weights, defects rate were also studied.

**Results:**

There was no difference in the baseline demographics between the blood type groups. There was a statistically significant positive association between the combination of female blood type AB and male blood type AB with biochemical pregnancy, clinical pregnancy, and live birth rate (OR 1.36; 95% CI, 1.05–1.78; *P* = 0.02 and OR 1.31; 95% CI, 1.0–1.68; *P* = 0.031 and OR 1.28; 95% CI, 1.01–1.63; *P* = 0.041 respectively). No statistically significant difference was observed between joint mother/father ABO blood types and high-quality embryo rate, early abortion rate, birth sex, birth weights, and birth defect rate.

**Conclusions:**

Our findings suggest that the success rate of IVF/ICSI cycles in parent mating AB blood type is higher than that in other blood type combination groups.

## Introduction

ABO blood group antigen is a complex molecule expressed on the surface of human red blood cells and some other cell types and various tissues. They play a major role in transfusion medicine, meanwhile, increasing evidence suggests that the ABO blood group is related to the development of many human diseases. A number of publications have studied the relation between blood group and female infertility including ovarian reserve, recurrent miscarriage, and live birth.

As early as 1960, some authors proposed that ABO blood incompatibility may be associated with infertility (1). In 1967 Schwimmer et al. suggested that ABO blood group incompatibility was one of the possible immunologic causes of infertility, they found that compared with couples with organic causes of infertility, couples with primary and secondary unexplained infertility possessed a higher incidence of ABO incompatibility. However, the association between ABO blood groups and infertility has been a point of controversy, while some studies support the absence of any relationship between blood groups and infertility in different populations. The correlation of blood groups to ovarian reserve has been investigated by multiple studies. Nejat et al. showed that blood type O was associated with a diminished ovarian reserve and that the A blood group antigen appears to be protective of ovarian reserve (2). On the contrary, Lin et al. (3) showed that Chinese women with blood type O had a less diminished ovarian reserve than women with blood types B and AB, who had more diminished ovarian reserve while blood type A was not associated with ovarian reserve. Other studies could not confirm this and show no correlation of blood groups to ovarian reserve response during *in vitro* fertilization (IVF) treatment (4–7). Recently, a systematic review and meta-analysis was conducted, which suggested that ABO blood groups are not associated with ovarian reserve, Ovarian hyperstimulation syndrome (OHSS), and outcomes of assisted reproductive technology (ART) (8).

Two retrospective studies on infertile women undergoing IVF indicated conflicting conclusions about whether live birth relates to ABO blood type (9, 10). Some studies suggest that ABO blood type has an impact on female infertility, which is related to the incidence of ovarian hyperstimulation syndrome and endometriosis, important influence factors of pregnancy. A predominance of blood group A was shown in patients with endometriosis by studying 231 women with endometriosis and 166 infertile women without endometriosis (11). One case-control study on 121 Caucasian patients showed a positive association between A blood group and early-onset ovarian hyperstimulation syndrome (12).

Despite the aforementioned findings, there is a lack of literature investigating joint parent ABO blood types in a large cohort. This study explores whether ABO blood type is associated with the clinical outcomes of IVF treatment, history of spontaneous miscarriage, embryo quality parameters, and birth sex, weight, and birth defect rates in Chinese infertile couples.

## Materials and Methods

### Study Population

**Figure 1:**
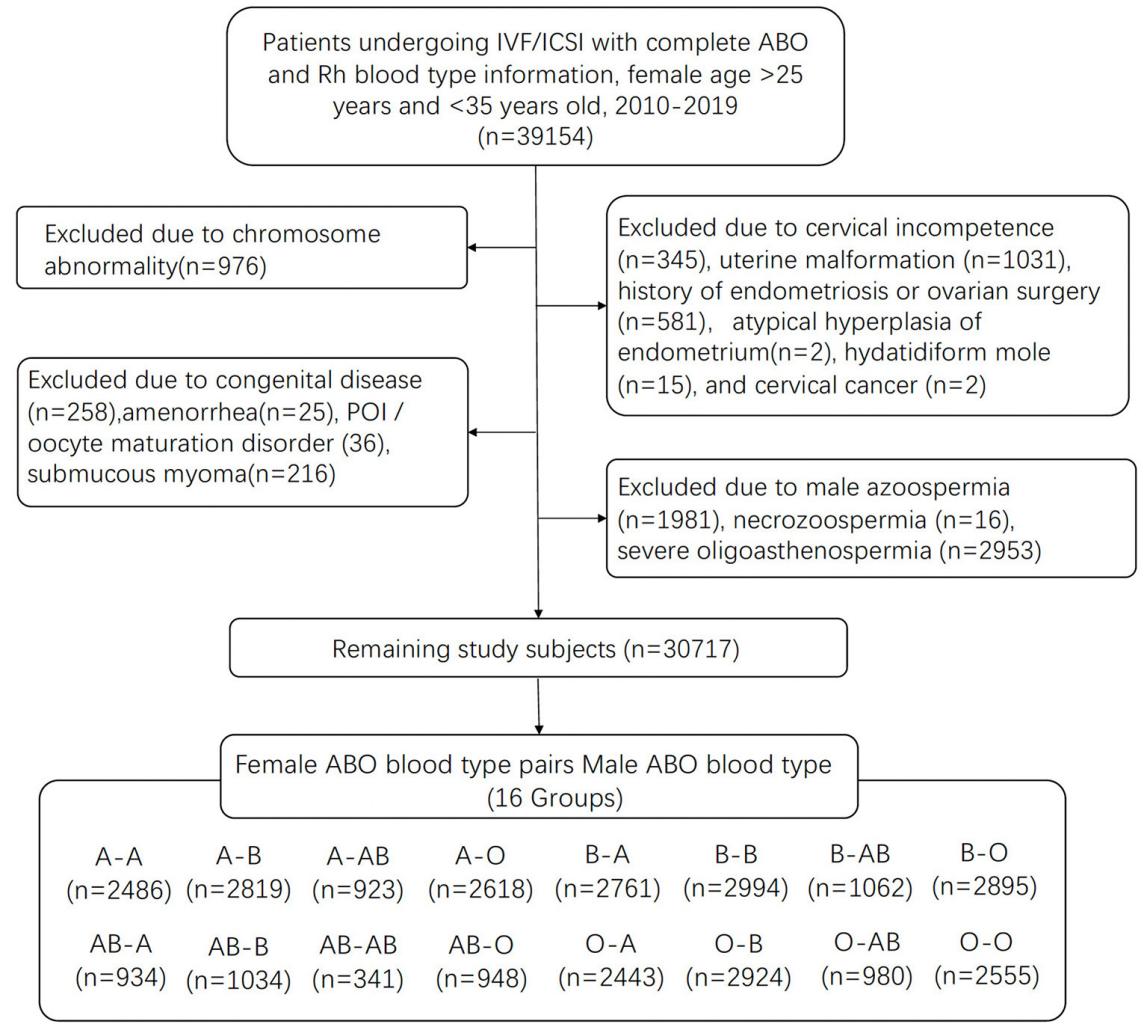
Study flowchart.

This retrospective cohort study was approved by the ethics committee of the First Affiliated Hospital of Zhengzhou University. Patients undergoing cycles of IVF/intracytoplasmic sperm injection (ICSI) at the Reproductive Medical Center, First Affiliated Hospital of Zhengzhou University, between January 2010 and December 2019 were included in this study. Follow-up data was finished in December 2020 and data took place in March 2021. From the initial pool of 39,477 cycles, 39,154 cycles have complete ABO and Rh blood type information with female patients aged >25 years and <35 years. Potential confounders may cause spontaneous abortion or affect ovarian response and ART outcomes were excluded in this study: chromosome abnormality (*n* = 976), cervical incompetence (*n* = 345), uterine malformation (*n* = 1,031), history of endometriosis or ovarian surgery (*n* = 581), atypical hyperplasia of endometrium (*n* = 2), hydatidiform mole (*n* = 15), and cervical cancer (*n* = 2), congenital disease (*n* = 258), amenorrhea (*n* = 25), POI/oocyte maturation disorder (36), submucous myoma (*n* = 216). Males with diagnoses of azoospermia (*n* = 1,981) and necrozoospermia (*n* = 16) were excluded. The blood type of donors of semen in artificial insemination (AID) or IVF with frozen semen donor (IVF-D) cannot be tracked. Males with severe oligoasthenospermia (*n* = 2,953) were also excluded to eliminate the influence of sperm quality on fertilization and ART outcomes. A total of 30,717 cycles met the inclusion criteria. Information on patients' characteristics, ART outcomes, and birth information for each couple were collected from the Clinical Reproductive Medicine Management System/Electronic Medical Record Cohort Database of the Reproductive Medical Center of the First Affiliated Hospital of Zhengzhou University Figure 1.

### Clinical and Laboratory Protocols

A blood type and screen were obtained in all patients as part of the initial infertility workup at the hospital's laboratory. Controlled ovarian hyperstimulation (COH), human chorionic gonadotropin (hCG) trigger, oocyte retrieval, embryo culture, and embryo transfer (ET) performed based on established protocols (13).

The use of GnRH-a differed in the two COH protocols: for GnRH-a prolonged protocol, 3.75 mg of GnRH-a was injected on day 2 of the menstrual cycle. For the short GnRH-a long protocol, 0.1 mg of GnRH-a was injected daily from the mid-luteal phase. When the patient achieved the criteria for pituitary suppression, Gonadotropin (Gonal-F, Merck, Germany; Puregon, Organon, Netherlands; Urofollitropin, Livzon, China) was used to initiate ovarian stimulation with the dose range of 75–300 IU based on the ovarian response (75–150 IU for normal or high ovarian response; 150–300 IU for reduced ovarian response). The exact dose of gonadotropin was adjusted based on age, body mass index (BMI, kg/m^2^), antral follicle count (AFC), and anti-Müllerian hormone (AMH) level of the individual patient. hCG (Livzon) was injected with the oocyte maturation trigger and was administered when at least two follicles had reached 18 mm. Oocytes were collected by a transvaginal ultrasound-guided puncture at 36–37 h after hCG injection. Progesterone in oil was used for luteal support at a dose of 60 mg per day after oocyte pick-up.

Based on the condition of semen and the couples' reproductive history, fertilization was carried out with short-time insemination or ICSI insemination (14). The embryo morphology in the cleavage stage was observed and graded according to Peter's standards (15) on day 3. Good quality embryos were defined when they graded better than II. Day 5 blastocyst-stage embryos were graded according to the Gardner standard (16). The condition and the willingness of the patients were comprehensively considered to decide whether to perform fresh ET or frozen-thawed embryo transfer (FET).

Based on the regularity of the menstrual cycle, FET cycles were divided into natural cycles and artificial cycles. For natural cycles, patients were allocated to undergo ultrasonic evaluation starting from day 8–9 of the menstrual cycle. When the diameter of the dominant follicle was 16–20 mm, a blood sample test for progesterone (P) and luteinizing hormone (LH) levels was conducted to monitor ovulation. Thawing and transferring were performed 3 days for cleavage-stage embryos or 5 days for blastocyst-stage embryos after ovulation. Intramuscular (im) progesterone (40 mg) starting on the day of ovulation and oral dydrogesterone (20 mg) starting on the embryo transfer day were used for luteal support. For artificial cycles, patients began oral estradiol [2 mg, Progynova; Bayer, Leverkusen, Germany] twice a day on cycle day 3. This dose was adjusted based on the endometrial thickness every 4 days. After 12–14 days, an ultrasound was performed and a serum progesterone level was determined. If no leading follicle was present, progesterone (60 mg, im) and oral dydrogesterone [10 mg [this dose was changed to 20 mg 2 days later]] would be added to the regimen.

The serum hCG biochemical pregnancy tests were performed 14 and 18 days after the embryo transfer. For patients with hCG was more than 50 IU/L, transvaginal ultrasound was performed 35 days after embryo transfer.

### Study Variables

Baseline demographics recorded for each patient included age (years), BMI (kg/m^2^), infertility diagnosis, ABO blood type, and rhesus factor type. The primary outcome of this study was the clinical outcomes of IVF treatment. Biochemical pregnancy was defined as an increase of HCG levels higher than a detailed range at 14 days after embryo transfer. Clinical pregnancy was defined as one or multiple gestational sac(s) and cardiac activity seen via ultrasound 35 days after embryo transfer. Live birth was defined as any birth event in which at least one baby is born alive. Early spontaneous miscarriage was defined as a pregnancy loss before 14 weeks of gestation following clinical pregnancy. Recurrent spontaneous abortion (RSA) was defined as the occurrence of at least two spontaneous losses before fetal viability.

### Statistical Analysis

Association analyses were performed using custom R scripts. Results for continuous data are given as mean ± standard deviation (SD). Results for categorical variables are given as the number of cases (*n*) with a percentage of occurrence (%). The proportions of the different blood types were compared by the chi-squared test. Analysis of variance (ANOVA) was used for parametric data between blood type groups. Previous spontaneous abortion, embryo quality parameters, ART outcomes, birth sex, weight (log2 transformed), and defects were compared by blood type groups using Multivariable logistic regression models while adjusting for potential confounders. The study population was divided into 16 groups with female ABO blood type pairs and male ABO blood types. The association between one specific blood type group and clinical outcomes were analyzed when all the other blood type group were used as controls. ORs with 95% CIs were estimated from the model. Three models were used to test spontaneous abortion association: (1) a dosage model, which was treated as recurrent miscarriage, marked 1 and as O; (2) a dominant model, which was treated 0 as absent and more than 1 abortion as present; and (3) a continuous model to study the number of spontaneous abortions. *P* < 0.05 was considered to be statistically significant. The Bonferroni correction was used for the *P*-value correction of multiple comparisons.

## Results

The distribution of the study cohort based on blood type was as follows: 8,846 (28.80%) females with blood type A; 3,257 (10.60%) females with blood type AB; 9,712 (31.62%) females with blood type B; and 8,902(28.98%) females with blood type O. For Rh blood type, 98 (0.032%) females with blood type Rh-; 30,619 (99.68%) females with blood type Rh+; This distribution of blood types were similar to the male participant results: 8,624 (28.08%) male participants with blood type A; 3,257 (10.76%) male participants with blood type AB; 9,771 (31.81%) male participants with blood type B; and 9,016 (29.35%) male participants with blood type O. For Rh blood type, 120 (0.039%) female participants with blood type Rh–; 330,597 (99.61%), female participants with blood type Rh+. As shown in Supplementary Table 1, there was no difference in the distribution of blood type between infertile female and male participants.

Demographic and clinical characteristics of the study population are shown in Table 1 and Supplementary Table 1. The women's average age was 30.0 ± 2.83 years. There was no difference in the age, BMI, duration of subfertility, primary subfertility, infertility diagnoses, basal sex hormone, COH and FET Protocols, peak estradiol level on the day of hCG trigger, peak endometrial thickness, number of retrieved oocytes, percentage of MII oocytes, good-quality embryos, number of embryos transferred, stage of embryos transferred by blood type in female groups. *P*-value of recombinant follicle stimulating hormone (rFSH) dosage (*P* = 0.049) and the percentages of fresh embryo transfer or frozen-thawed embryo transfer (*P* = 0.046) were < 0.05, but there was no statistical difference when the two groups were compared.

**Table 1:** Overall demographics and patient characteristics of the study population stratified by female blood type (*n* = 30,717).

**Characteristics**	**All**	**A (***n*** = 8,846)**	**B (***n*** = 9,712)**	**AB (***n*** = 3,257)**	**O (***n*** = 8,902)**	***P*** **-value**
Age (y)*	30.06 ± 2.83	30.09 ± 2.84	30.06 ± 2.85	29.98 ± 2.82	30.05 ± 2.81	0.236
BMI (kg/m^2^)*	22.83 ± 3.22	22.82 ± 3.20	22.83 ± 3.19	22.91 ± 3.36	22.82 ± 3.20	0.554
Duration of subfertility (m)*	48.22 ± 33.58	48.47 ± 33.31	48.36 ± 34.09	47.68 ± 32.06	48.03 ± 33.83	0.613
Primary subfertility (%)^#^	44.93% (13,801/30,717)	45.16% (3,995/8,846)	45.53% (4,422/9,712)	45.26% (1,474/3,257)	43.92% (3,910/8,902)	0.145
Infertility diagnoses (%)^#^						0.056
Anovulation	12.79% (3,930/30,717)	13.60% (1,203/8,846)	12.26% (1191/9,712)	13.57% (442/3,257)	12.29% (1,094/8,902)	
Tubal factor	42.25% (12,979/30,717)	42.47% (3,757/8,846)	41.85% (4,064/9,712)	41.17% (1341/3,257)	42.88% (3,817/8,902)	
Male factor	3.16% (972/30,717)	3.18% (281/8846)	3.22% (313/9712)	2.86% (93/3257)	3.20% (285/8,902)	
Unexplained	29.41% (9034/30717)	29.11% (2,575/8,846)	29.73% (2,887/9,712)	29.92% (968/3,257)	29.25% (2,604/8,902)	
Combined	12.38% (3,802/30,717)	11.64% (1030/8846)	12.94% (1,257/9,712)	12.68% (413/3,257)	12.34% (1,102/8,902)	
Basal FSH (IU/L)*	6.645 ± 2.32	6.923 ± 2.28	6.958 ± 2.22	6.827 ± 2.12	6.983 ± 2.52	0.085
Basal LH (IU/L)*	4.810 ± 3.53	4.840 ± 3.55	4.75 ± 3.54	4.82 ± 3.52	4.83 ± 3.50	0.708
Basal T (ng/mL)*	0.26 ± 0.25	0.26 ± 0.22	0.27 ± 0.29	0.26 ± 0.23	0.26 ± 0.25	0.060
Basal E2(pg/mL)*	25.86 ± 32.73	26.02 ± 31.53	26.02 ± 33.22	35.86 ± 32.97	25.47 ± 33.26	0.956
Basal P (ng/mL)*	0.30 ± 0.70	0.30 ± 0.66	0.30 ± 0.74	0.30 ± 0.68	0.30 ± 0.69	0.427
COH/FET Protocols (%)^#^						0.299
Short GnRH-a long protocol	24.80% (7,617/30,717)	24.34% (2,153/8,846)	24.80% (2,409/9,712)	24.07% (784/3,257)	25.51% (2,271/8,902)	
GnRH-a prolonged protocol	31.95% (9,813/30,717)	31.82% (2,815/8,846)	31.41% (3,051/9,712)	32.32% (1,056/3,257)	32.48% (2,891/8,902)	
Natural cycle	14.56% (4,472/30,717)	14.44% (1,277/8,846)	14.52% (1,410/9,712)	14.95% (487/3,257)	14.58% (1,298/8,902)	
Artificial cycle	28.70% (8,815/30,717)	29.40% (2,601/8,846)	29.26% (2,842/9,712)	28.55% (930/3,257)	27.43% (2,442/8,902)	
Dosage of rFSH (IU)*	2150 ± 649.72	2142 ± 657.90	2152 ± 653.65	2112.1 ± 644.34	2169 ± 639.14	0.049^a^
Peak E2 level on day of hCG trigger (pg/mL)*	3890.4 ± 2238.8	3885.4 ± 2232.3	3896.9 ± 2217.06	3930.4 ± 2257.78	3874.0 ± 2261.55	0.956
Peak endometrial thickness (mm)*	12.20 ± 2.42	12.24 ± 2.41	12.15 ± 2.41	12.21 ± 2.44	12.21 ± 2.44	0.157
Number of retrieved oocytes (*n*)*	12.56 ± 5.66	12.60 ± 5.63	12.43 ± 5.66	12.84 ± 5.56	12.59 ± 5.77	0.063
Percentage of MII oocytes (%)	0.817 ± 0.16	0.818 ± 0.16	0.817 ± 0.16	0.819 ± 0.16	0.816 ± 0.16	0.159
Good-quality embryos (%)^#^	0.67 ± 0.27	0.672 ± 0.26	0.667 ± 0.26	0.675 ± 0.26	0.671 ± 0.27	0.587
The Number of ET (*n*)*	1.79 ± 0.51	1.80 ± 0.52	1.79 ± 0.51	1.78 ± 0.52	1.78 ± 0.51	0.211
Stage of embryos Transferred (%)^#^						0.415
Cleavage stage	73.46% (22,566/30,717)	73.78% (6,527/8,846)	73.03% (7,093/9,712)	72.80% (2,371/3,257)	73.86% (6,575/8,902)	
Blastocyte stage	26.54% (8,151/30,717)	26.22% (2,319/8,846)	26.97% (2,619/9,712)	27.20% (886/3,257)	26.14% (2,327/8,902)	
Percentages of fresh ET or FET (%)^#^						0.046^a^
Fresh ET	56.74% (17,430/30,717)	56.16% (4,968/8,846)	56.22% (5,460/9,712)	56.49% (1,840/3,257)	57.99% (5,162/8,902)	
FET	43.26% (13,287/30,717)	43.84% (3,878/8,846)	43.78% (4,252/9,712)	43.51% (1,417/3,257)	42.01% (3,740/8,902)	

* *Indicates continuous variables, presented as mean ± standard deviation (SD). ^#^Indicates categorical variables, presented as percentage (number)*.

a *Indicates P < 0.05, but no significant difference between each group. BMI, body mass index; FSH, follicle-stimulating hormone; LH, luteinizing hormone; T, testosterone; E2, estradiol; P, progesterone; COH, controlled ovarian hyperstimulation; FET, frozen-thawed embryo transfer; rFSH, recombinant follicle stimulating hormone; ET, embryo transfer*.

Given the small sample size of patients with negative Rh in the population, especially when it was split into the four ABO blood groups, the rhesus factor was not included in the downstream association test.

### History of Spontaneous Miscarriage Association Analysis

Potential interaction between the ABO blood group of mother/father mating and the history of spontaneous miscarriage were investigated by three models as described before. There was a statistically significantly higher percentage of the combination of female blood type AB and male blood type O in couples with at least one spontaneous miscarriage history (odds ratio [OR] 1.03; 95% confidence interval [CI], 1–1.05; *P* = 0.0234) than for the rest. However, no statistically significant difference was observed in Dosage and Continuous models (OR 1.01; 95% CI, 1–1.01; *P* = 0.243 and OR 1.03; 95% CI, 1–1.06; *P* = 0.08, respectively), as shown in Table 2.

**Table 2:** Multivariate logistic regression analyses between the history of spontaneous miscarriage and parental blood type combinations.

**Female blood type**	**Male blood type**	**Dosage model**		**Dominant model**		**Continuous model**	
		**OR (95%CI)**	***P*** **-value**	**OR (95%CI)**	***P*** **-value**	**OR (95%CI)**	***P*** ** value**
AB	B	1.00 (0.99–1.01)	0.874	0.99 (0.97–1.01)	0.433	0.99 (0.96–1.02)	0.384
AB	A	1.00 (0.99–1.01)	0.753	0.99 (0.97–1.01)	0.348	1.00 (0.97–1.03)	0.843
AB	O	1.01 (1.00–1.01)	0.243	1.03 (1.00–1.05)	0.023	1.03 (1.00–1.06)	0.080
AB	AB	0.99 (0.98–1.01)	0.304	0.99 (0.96-1.02)	0.426	0.98 (0.94–1.02)	0.323
B	B	1.00 (0.99–1.00)	0.521	1.00 (0.99–1.01)	0.871	0.99 (0.97–1.01)	0.438
B	A	1.00 (0.99–1.01)	0.966	1.01 (0.99–1.02)	0.262	1.01 (0.99–1.03)	0.213
B	O	1.00 (1.00–1.01)	0.443	0.99 (0.98–1.01)	0.214	0.99 (0.98–1.01)	0.566
B	AB	1.00 (0.99–1.01)	0.963	1.00 (0.98–1.03)	0.646	1.00 (0.98–1.03)	0.802
A	B	1.00 (1.00–1.01)	0.465	1.01 (0.99–1.02)	0.433	1.02 (1.00–1.04)	0.113
A	A	1.00 (0.99–1.00)	0.467	0.99 (0.98–1.01)	0.355	0.99 (0.97–1.01)	0.166
A	O	1.00 (0.99–1.00)	0.525	1.00 (0.98–1.01)	0.561	0.99 (0.97–1.01)	0.388
A	AB	1.00 (1.00–1.01)	0.373	1.01 (0.99–1.03)	0.308	1.01 (0.98–1.04)	0.370
O	B	1.00 (0.99–1.01)	0.993	1.00 (0.99–1.01)	0.910	1.00 (0.98–1.02)	0.816
O	A	1.00 (1.00–1.01)	0.321	1.00 (0.99–1.02)	0.676	1.00 (0.98–1.02)	0.806
O	O	1.00 (0.99–1.00)	0.356	1.00 (0.99–1.02)	0.731	1.00 (0.98–1.02)	0.767
O	AB	1.00 (0.99–1.01)	0.885	0.99 (0.97–1.01)	0.348	0.99 (0.96–1.02)	0.635

### Embryo Quality Parameters Association Analysis

Parent blood type combinations and embryo quality parameters, including fertilization rate, cleavage rate, and high-quality embryo rate were assessed by adjusting controlled ovarian hyperstimulation protocols. The combination of female blood type B and male blood type A has a negative association with fertilization rate (OR 0.98; 95% CI, 0.97–1; *P* = 0.01), while the combination of female blood type O and male blood type A has a slightly negative association with cleavage rate (OR 1; 95% CI, 0.99–1; *P* = 0.027). As evident in Table 3, no statistically significant difference was observed between the ABO blood group of mother/father mating blood types and High-Quality Embryo Rate.

**Table 3:** Multivariate logistic regression analyses between embryo quality parameters and parental blood type combinations by adjusting for controlled ovarian hyperstimulation protocols.

**Female blood type**	**Male blood type**	**Fertilization rate**		**Cleavage rate**		**High quality embryo rate**	
		**OR (95%CI)**	***P*** **-value**	**OR (95%CI)**	***P*** **-value**	**OR (95%CI)**	***P*** **-value**
B	A	0.98 (0.97–1.00)	0.010	1.00 (1.00–1.01)	0.251	0.99 (0.97–1.01)	0.354
B	O	1.01 (0.99–1.02)	0.245	1.00 (1.00–1.00)	0.942	1.02 (1.00–1.04)	0.110
B	B	1.01 (0.99–1.02)	0.315	1.00 (1.00–1.00)	0.783	0.99 (0.97–1.01)	0.442
B	AB	1.01 (0.98–1.03)	0.563	1.00 (0.99–1.00)	0.254	1.00 (0.97–1.03)	0.843
AB	A	1.00 (0.98–1.02)	0.813	1.00 (1.00–1.01)	0.343	0.99 (0.96–1.02)	0.564
AB	O	1.00 (0.98–1.03)	0.854	1.00 (0.99–1.00)	0.386	1.01 (0.98–1.04)	0.520
AB	B	1.00 (0.98–1.02)	0.847	1.00 (0.99–1.01)	0.818	1.01 (0.98–1.04)	0.529
AB	AB	1.01(0.97–1.04)	0.701	1.00 (0.99–1.01)	0.832	0.98 (0.94–1.02)	0.313
A	A	1.01 (0.99–1.02)	0.278	1.00 (1.00–1.01)	0.654	1.01 (0.99–1.03)	0.510
A	O	1.00 (0.99–1.02)	0.839	1.00 (0.99–1.00)	0.464	0.98 (0.96–1.00)	0.061
A	B	0.99 (0.98–1.01)	0.282	1.00 (1.00–1.00)	0.992	1.01 (0.99–1.03)	0.308
A	AB	1.00 (0.97–1.02)	0.782	1.00 (1.00–1.01)	0.658	1.00 (0.97–1.03)	0.816
O	A	1.01 (1.00–1.03)	0.089	1.00 (0.99–1.00)	0.027	1.01 (0.99–1.03)	0.497
O	O	0.99 (0.97–1.00)	0.127	1.00 (1.00–1.01)	0.171	1.00 (0.98–1.02)	0.860
O	B	1.00 (0.99–1.02)	0.840	1.00 (1.00–1.00)	0.680	0.99 (0.98–1.01)	0.508
O	AB	0.99 (0.97–1.02)	0.565	1.00 (1.00–1.01)	0.559	1.00 (0.98–1.03)	0.808

### Assisted Reproductive Technology (ART) Outcomes Association Analysis

Outcomes of treatment to blood groups, including Biochemical and clinical Pregnancy, early abortion rate, and live birth rate were further compared. As shown in Table 4, there was a statistically significant positive association between the combination of female blood type AB and male blood type AB with biochemical pregnancy, clinical pregnancy, and live birth rate (OR 1.36; 95% CI, 1.05–1.78; *P* = 0.02 and OR 1.31; 95% CI, 1.03–1.68; *P* = 0.031 and OR 1.28; 95% CI, 1.01–1.63; *P* = 0.041, respectively). No statistically significant difference was observed between couple combinations for ABO blood groups and early abortion rate.

**Table 4:** Multivariate logistic regression analyses between assisted reproductive technology (ART) outcomes and parental blood type combinations with adjustment for the number of transfer embryo(s).

**Female blood type**	**Male blood type**	**Biochemical pregnancy**		**Clinical pregnancy**		**Early abortion rate**		**Live birth rate**	
		**OR (95%CI)**	***P*** **-value**	**OR (95%CI)**	***P*** **-value**	**OR (95%CI)**	***P*** **-value**	**OR (95%CI)**	***P*** **-value**
AB	B	1.04 (0.88–1.23)	0.614	0.93 (0.79–1.09)	0.374	0.87 (0.69–1.10)	0.255	0.91 (0.78–1.06)	0.234
AB	A	0.92 (0.78–1.10)	0.361	0.98 (0.83–1.16)	0.824	1.08 (0.87–1.35)	0.479	0.96 (0.82–1.13)	0.646
AB	O	0.90 (0.76–1.07)	0.239	0.97 (0.83–1.14)	0.742	1.04 (0.83–1.30)	0.708	1.02 (0.87–1.20)	0.769
AB	AB	1.36 (1.05–1.78)	0.020	1.31 (1.03–1.68)	0.031	1.01 (0.71–1.42)	0.975	1.28 (1.01–1.63)	0.041
B	B	0.99(0.89–1.10)	0.841	0.98 (0.88–1.09)	0.749	1.19 (0.94–1.50)	0.155	0.99 (0.90–1.10)	0.920
B	A	0.97 (0.86–1.09)	0.585	1.00 (0.90–1.11)	0.972	0.83 (0.66–1.05)	0.119	0.99 (0.89–1.10)	0.809
B	O	1.06 (0.95–1.19)	0.305	1.05 (0.94–1.17)	0.383	0.96 (0.76–1.21)	0.738	1.07 (0.96–1.19)	0.222
B	AB	0.97(0.82–1.14)	0.678	0.94 (0.80–1.10)	0.458	1.17 (0.81–1.65)	0.395	0.90 (0.77–1.05)	0.196
A	B	0.99 (0.88–1.11)	0.861	1.03 (0.93–1.15)	0.572	1.01 (0.71–1.42)	0.959	1.01 (0.91–1.13)	0.802
A	A	1.05 (0.93–1.18)	0.435	0.99 (0.88–1.10)	0.806	1.13 (0.81–1.56)	0.477	1.03 (0.93–1.15)	0.556
A	O	0.97 (0.86–1.08)	0.550	0.96 (0.86–1.08)	0.515	0.82 (0.58–1.16)	0.275	0.92 (0.83–1.03)	0.137
A	AB	1.00 (0.84–1.19)	0.981	1.04 (0.88–1.23)	0.623	1.15 (0.68–1.86)	0.593	1.09 (0.92–1.28)	0.315
O	B	1.00 (0.90–1.12)	0.962	1.02 (0.92–1.14)	0.690	0.97 (0.76–1.23)	0.781	1.04 (0.93–1.15)	0.500
O	A	1.03 (0.91–1.15)	0.680	1.02 (0.92–1.15)	0.675	1.04 (0.83–1.31)	0.714	1.00 (0.89–1.11)	0.971
O	O	1.02 (0.91–1.14)	0.739	1.00 (0.89–1.11)	0.966	1.08 (0.86–1.37)	0.490	1.00 (0.9–1.120)	0.980
O	AB	0.90 (0.77–1.07)	0.242	0.91 (0.77–1.07)	0.240	0.78 (0.53–1.13)	0.201	0.92 (0.79–1.08)	0.303

### Birth Sex, Weights, Defects Rate Association Analysis

Finally, a multivariate logistic regression analysis was performed to determine the relationship between blood type and birth sex, birth weights, and birth defects rate in single live born cycles. As shown in Table 5, no blood type was statistically significantly associated with birth sex, birth weight, and birth defect rate.

**Table 5:** Multivariate logistic regression analyses between birth sex, birth weights, birth defects, and parental blood type combinations in single live born cycles.

**Female blood type**	**Male blood type**	**Birth sex**		**Birth weights**		**Birth defects**	
		**OR (95%CI)**	***P*** **-value**	**OR (95%CI)**	***P*** **-value**	**OR (95%CI)**	***P*** **-value**
AB	B	0.94 (0.72–1.22)	0.630	1.00 (0.98–1.02)	0.936	0.80 (0.34–1.75)	0.598
AB	A	1.02 (0.78–1.34)	0.864	1.00 (0.98–1.02)	0.832	1.64 (0.78–3.38)	0.182
AB	O	0.95 (0.73–1.24)	0.698	1.00 (0.98–1.02)	0.898	0.89 (0.39–1.90)	0.764
AB	AB	1.19 (0.82–1.73)	0.365	1.01 (0.98–1.04)	0.528	0.61 (0.14–1.90)	0.447
B	B	1.18 (0.99–1.41)	0.059	1.01 (0.99–1.03)	0.571	0.72 (0.37–1.32)	0.297
B	A	1.02 (0.85–1.22)	0.853	1.01 (0.99–1.03)	0.402	1.00 (0.55–1.80)	0.994
B	O	0.85 (0.71–1.01)	0.064	0.99 (0.97–1.01)	0.319	1.55 (0.86–2.78)	0.143
B	AB	0.95 (0.74–1.23)	0.696	0.99 (0.96–1.02)	0.546	0.73 (0.26–1.79)	0.510
A	B	0.96 (0.80–1.14)	0.630	1.01 (0.98–1.04)	0.479	1.19 (0.65–2.14)	0.558
A	A	0.86 (0.72–1.04)	0.117	1.01 (0.98–1.04)	0.517	0.71 (0.38–1.29)	0.274
A	O	1.14 (0.95–1.37)	0.166	0.98 (0.95–1.01)	0.211	0.92 (0.50–1.68)	0.799
A	AB	1.14 (0.88–1.49)	0.322	1.00 (0.96–1.04)	0.872	1.63 (0.69–3.71)	0.250
O	B	0.90 (0.76–1.08)	0.264	0.99 (0.97–1.01)	0.336	1.26 (0.71–2.21)	0.419
O	A	1.12 (0.94–1.35)	0.209	0.99 (0.97–1.01)	0.306	1.01 (0.57–1.77)	0.967
O	O	1.08 (0.90–1.30)	0.409	1.02 (1.00–1.04)	0.054	0.76 (0.41–1.36)	0.363
O	AB	0.84 (0.65–1.10)	0.203	1.00 (0.97–1.03)	0.918	1.04 (0.42–2.36)	0.935

## Discussion

To the best of our knowledge, this study represents the largest retrospective cohort study evaluating the association between parent ABO blood group pairs and ART outcomes published so far. The result suggests there was a statistically significant positive association between the combination of female blood type AB and male blood type AB with biochemical pregnancy, clinical pregnancy, and live birth rate. No statistically significant difference was observed between couple combinations for ABO blood groups and High-quality embryo rate, early abortion rate, birth sex, birth weights, and birth defect rate.

As early as 1943, Levine identified ABO incompatibility as a cause of early abortions and stillbirths. By analyzing the relation between mother/father joint ABO blood group in 79 couples suffering from recurrent abortion in India, Malekasgar et al. show an excess of joint “A/B” blood groups in couples with RSA (17). However, this study presented the difference simply by the percentage of blood types not the *P*-value of statistical analysis. By screening the ABO blood groups in aborted fetuses and their parents in 124 early spontaneous abortions, Bandyopadhyay found a significantly higher (*P* < 0.05) frequency of ABO incompatibility in couples with miscarriage when compared with newborns and their parents from the same area (18). This conclusion indicates that the ABO incompatibility between the father and mother may be a risk factor for early spontaneous abortions. However, these results lacked statistical power to detect associations of blood groups with history of spontaneous abortions before ART treatment, or early in spontaneous abortions following IVF-ET.

Awartani et al. (7) compared the clinical parameters of 566 IVF treatment cycles with different ABO blood types and found no significant association between blood type and pregnancy rate. This result agrees with another retrospective study (19) that evaluated the effect of non-O blood type on clinical pregnancy of 497 women and found no statistically significant association between them. For live birth, two retrospective studies on infertile women undergoing IVF came to different conclusions. One study in a cohort of 626 infertile women suggested that women with blood type B had a significantly higher likelihood of live birth following IVF-ET (9). However, Pereira et al. observed no relation of blood groups to live-birth rate by assessing 2,329 patients undergoing fresh IVF with day 5 single embryo transfer (10). In our study population, couples who have joint “AB/AB” blood types had a significantly higher likelihood of biochemical pregnancy, clinical pregnancy, and live birth. Using the ABO incompatibility effect infertility hypothesize is easy to explain our findings, considering that the same blood types were completely compatible and no antibodies were activated to attack each other.

No association between ABO blood type and birth weight at delivery was found in our study, which reflects the conclusions of Pereira (10). In 1975, Allan reported that the birth sex ratio of male to female babies is related to the ABO blood group of babies and mothers by studying 53,679 mother-baby combinations. AB mothers were found to have higher sex ratio babies while A babies have a lower sex ratio (20). The authors suggest that these differences may be caused by the interaction of the ABO genes and some unknown sex-determining genes with estrogen and progesterone. No correlation was found between blood type and birth sex in our cohort.

As mentioned before, the current evidence available is not sufficient to confirm that the blood type ABO is related to some aspects of female fertility. The underlying mechanisms of ABO blood type incompatibility may play some role in abortion. Some authors hypothesized that ABO blood type may result in infertility because of the presence of incompatible antibodies of spermatozoa in the serum or the secretions of the mother's genital tract. Genetics is another possible mechanism, given the candidate *NR5A1* and *TGFBR1* genes impacting oocyte quality or early implantation are in proximity to the 9q34 locus of the ABO gene (21). The other possible mechanism of ABO-related infertility was its effect on thrombosis, some studies suggested the formation of blood vessels at the maternal-fetal interface may lead to the failure of implantation or placenta, further influent the clinical outcomes of IVF (22). The relationship between non-O blood type and venous thromboembolism has been observed in previous studies (23) but data remains controversial (24). In consideration of the maternal-fetal differences in ABO membrane protein structure, studies on couple combinations for ABO blood groups were not sufficient to address the underlying mechanisms.

This study design has a number of limitations (observational, non-randomized, and retrospective). AMH level is missing for patients collected between January 2010 and December 2019. Most of them did not receive serum AMH testing, as this clinical test has only been used in our hospital since 2017. It is noteworthy that newborn blood type information is missing, and our results of no association between blood type and birth ratio do not exclude other possibilities. The mechanisms that could explain joint “AB/AB” blood types having higher success rates are not entirely clear. Thus, further prospective and mechanism studies are needed to prove our results. Nonetheless, our findings relating mother/father joint ABO blood group to clinical outcomes of IVF/ICSI, indicate the success rate of IVF/ICSI cycles in parent mating AB blood type is higher than that in other blood type combination groups. Our results provide evidence of the relationship between joint ABO blood group and IVF/ICSI clinical outcomes in a Chinese population.

## Conclusion

In conclusion, the current large sample size retrospective cohort study demonstrated the statistically significant positive association between the combination of female blood type AB and male blood type AB with biochemical pregnancy, clinical pregnancy, and live birth rate. Couples who have joint “AB/AB” blood types had a significantly higher likelihood of success rate for IVF/ICSI cycles.

## Data Availability Statement

The original contributions presented in the study are included in the article/Supplementary Material, further inquiries can be directed to the corresponding author/s.

## Ethics Statement

This study was approved by the Ethics Committee of the First Affiliated Hospital of Zhengzhou University. The studies involving human participants were reviewed and approved by the Ethics Committee of the First Affiliated Hospital of Zhengzhou University. The patients/participants provided their written informed consent to participate in this study.

## Author Contributions

XB and YS designed the study. XB, FZ, and HS analyzed the data. XB, ZB, and YL wrote the manuscript. YS revised the manuscript. All authors contributed to the article and approved the submitted version.

## Funding

This work was funded by the International (Regional) Cooperation and Exchange (ICE) Projects of the National Natural Science Foundation of China (NSFC) (FDN-81820108016 to YS), the Joint Construction Project of the Key Project of Medical Science and Technology of Henan Province of China (LHGJ20190121 to XB), and the Projects of the National Science Fund for Young Scholars (82001529 to FZ).

## Conflict of Interest

The authors declare that the research was conducted in the absence of any commercial or financial relationships that could be construed as a potential conflict of interest.

## Publisher's Note

All claims expressed in this article are solely those of the authors and do not necessarily represent those of their affiliated organizations, or those of the publisher, the editors and the reviewers. Any product that may be evaluated in this article, or claim that may be made by its manufacturer, is not guaranteed or endorsed by the publisher.
