# Supplementary material for: Parent Joint AB Blood Group Is Associated With Clinical Outcomes of in vitro Fertilization and Intracytoplasmic Sperm Injection Treatment in Chinese Women
Source: Front Med (Lausanne). 2022 May 4;9:813781. doi: 10.3389/fmed.2022.813781 (PMC9115895; doi:10.3389/fmed.2022.813781)
Supplement: Supplementary file 1 [file Table_1.DOCX]

**Supplemental Table 1**

**Overall ABO and Rh blood Type distributions in female and males undergoing cycles of IVF/ICSI.**

| **Blood Type** | | **Females** | **Males** | ***P* value** |
| --- | --- | --- | --- | --- |
| **Rh** | - | 0.032% (98/30717) | 0.039% (120/30717) | 0.136 |
|  | + | 99.68% (30619/30717) | 99.61% (30597/30717) |  |
| **ABO** | A | 28.80% (8846/30717) | 28.08% (8624/30717) | 0.252 |
|  | AB | 10.60% (3257/30717) | 10.76% (3306/30717) |  |
|  | B | 31.62% (9712/30717) | 31.81% (9771/30717) |  |
|  | O | 28.98% (8902/30717) | 29.35% (9016/30717) |  |
